# 

**Published:** 1871-02

**Authors:** 


### INDEX OF SUBJECTS.

ORIGINAL ARTICLES AND SPECIAL SELECTIONS:

Fulton County Medical Society........................................................45
Radical Cure of Artificial Anus—Treatment of Gonorrhœa wih Tannin and Glycerine.......................................................................48
Supposed Poisoning by Hydrate of Chloral, ........................................49
Therapeutics of Chronic Constipation—Sulphite of Soda.. ........................50
Shoulder-Tip Pain in Hepatic Diseases............................................51
Is it Proper for Consumptives to Marry ?.... .......................................53
Intra-Uterine Medication..............................................................56
Case of Cholera-Infantum with Anuria for five days..............................59
Substitute for Quinine.................................................................60

EXTRACTS AND GLEANINGS.

Retracted Nipple—Itch Treated by Balsam of Peru—Sulphite of Soda in Tinea Cahitis—61; Flatulent Distension of the Colon with or without Diarrhœa—Treatment of Constipation—62; Opium in Diabetes—Chloroform, pro et con—63; Large doses of Opium in Tetanus—The Pathology and Treatment of Scarlet Fever—64; Oxalate of Cerium in Dyspeptic Vomiting—Typhoid Fever—Atomized Medication for Asthma—Neuralgic Linament—65; Prescriptions for Whooping-Cough, Catarrhus Vessicæ, Diarrhœa of Children, Obstinate Vomiting, etc.—66; Treatment of Asthma, Chronic Dysentery, Nervous affections of the Abdominal Organs in Woman, etc.—67; Carbolic Acid in Eczema—Carbolated Alcohol—Iodized Syrup of Horse-Radish—Ergot of Rye in Neuralgia—Ergot in Puppera—Uterine Hemorrhage—68; Prophylaxis of Scarlet Fever and Measles—Sub. for Dover's Powders—Treatment for Tape Worm—Strumous Enlargement of the Lymphatics—69; Use of Sarsaparilla in Syphilis—Treatment of Infantile Eresypelas—70; Sulphate of Manganese in Chorea—Subnitrate of Bismuth in Diarrhœa—Sulphate of Nickle in Neuralgia—The use of Pepsin—71; Induction of Premature Labor—Treatment for Ingrowing Toe-Nails—72; Belladonna in arresting the Lacteal Secretion—Calabar Bean in Tetanus—Lobelia for Rigid Os Uteri—73; Carbolic Acid in Rubeola—Sulphurous Acid in Pyrosis—Digitalis in Menorrhagia—Simple Method of arresting Obstinate Epistoxis, 74; Bromide of Potassium for Infants—Tonics in Chronic Bronchitis—Cholera, 75;—Remedy for Whooping Cough—Treatment for Chora, 76.

BIOGRAPHICAL, ETHICAL, HYGENIC AND MISCELLANEOUS.

Our New Department—Biographical Sketch of A. E. Petticolas, 78; Correspondence, 79; Compensation, 83; Air, Water, Sewerage ..nd Drainage, 84; Fainting Fits and how to cure them—Perishing for Lack of Knowledge, 86; Bad Breath—Cleansing Blankets, 87; Look to your Pumps---Tight Lacing, 88; Perspiration---Thraldom of Fashion---Goodness better than Greatness, 89; Water-Drinking in Hot Weather---Music for Invalids---Sewing Machines and Health---Exercise before Breakfast, 90.

EDITORIALS, REVIEWS, ITEMS, AND NEWS.

Professional Unity---Medical Societies,..............................................91
Science and Peace,......................................................................94
The Albany Medical College............................................................96
Chloroform.................................................................................98
Please Excuse Us---Errata.............................................................100

